# Complete Chloroplast Genome Analysis of Two Important Medicinal *Alpinia* Species: *Alpinia galanga* and *Alpinia kwangsiensis*

**DOI:** 10.3389/fpls.2021.705892

**Published:** 2021-12-15

**Authors:** Yue Zhang, Mei-Fang Song, Yao Li, Hui-Fang Sun, Dei-Ying Tang, An-Shun Xu, Cui-Yun Yin, Zhong-Lian Zhang, Li-Xia Zhang

**Affiliations:** Yunnan Key Laboratory of Southern Medicine Utilization, Yunnan Branch of Institute of Medicinal Plant Development, Chinese Academy of Medical Sciences, Peking Union Medical College, Jinghong, China

**Keywords:** *Alpinia galanga*, *Alpinia kwangsiensis*, chloroplast genome, Zingiberaceae, phylogenetic relationship

## Abstract

Most *Alpinia* species are valued as foods, ornamental plants, or plants with medicinal properties. However, morphological characteristics and commonly used DNA barcode fragments are not sufficient for accurately identifying *Alpinia* species. Difficulties in species identification have led to confusion in the sale and use of *Alpinia* for medicinal use. To mine resources and improve the molecular methods for distinguishing among *Alpinia* species, we report the complete chloroplast (CP) genomes of *Alpinia galanga* and *Alpinia kwangsiensis* species, obtained via high-throughput Illumina sequencing. The CP genomes of *A. galanga* and *A. kwangsiensis* exhibited a typical circular tetramerous structure, including a large single-copy region (87,565 and 87,732 bp, respectively), a small single-copy region (17,909 and 15,181 bp, respectively), and a pair of inverted repeats (27,313 and 29,705 bp, respectively). The guanine–cytosine content of the CP genomes is 36.26 and 36.15%, respectively. Furthermore, each CP genome contained 133 genes, including 87 protein-coding genes, 38 distinct tRNA genes, and 8 distinct rRNA genes. We identified 110 and 125 simple sequence repeats in the CP genomes of *A. galanga* and *A. kwangsiensis*, respectively. We then combined these data with publicly available CP genome data from four other *Alpinia* species (*A. hainanensis*, *A. oxyphylla*, *A. pumila*, and *A. zerumbet*) and analyzed their sequence characteristics. Nucleotide diversity was analyzed based on the alignment of the complete CP genome sequences, and five candidate highly variable site markers (*trnS-trnG*, *trnC-petN*, *rpl32-trnL*, *psaC-ndhE*, and *ndhC-trnV*) were found. Twenty-eight complete CP genome sequences belonging to Alpinieae species were used to construct phylogenetic trees. The results fully demonstrated the phylogenetic relationship among the genera of the Alpinieae, and further proved that *Alpinia* is a non-monophyletic group. The complete CP genomes of the two medicinal *Alpinia* species provides lays the foundation for the use of CP genomes in species identification and phylogenetic analyses of *Alpinia* species.

## Introduction

*Alpinia* Roxb. is an important genus of Zingiberaceae and includes 250 species mainly distributed in tropical Southeast Asia, but their distribution extends south into Australia and the South Pacific islands, and west into India (Wu and Larsen, 2000; Wu et al., 2016; Li D. M. et al., 2020). There are approximately 50 species in China, mainly in the south (Wu and Larsen, 2000). Most *Alpinia* species are valued as foods, ornamental plants, or plants with medicinal properties. As such, studies of *Alpinia* species have focused on their effective chemical components and pharmacological properties. For example, *Alpinia oxyphylla* is an important food and traditional Chinese herbal medicine; previous studies have demonstrated its antioxidative, anti-inflammatory, anti-apoptotic, and neuropharmacological effects (Zhang et al., 2011; Gao et al., 2019; Li J. et al., 2020). *A. galanga* and *A. zerumbet* have been used as traditional medicines and food seasonings for hundreds of years (Chouni and Paul, 2018; Xiao et al., 2020). The rhizomes of *A. kwangsiensis* are used in Chinese traditional medicine to treat abdominal pain, stomach flu, vomiting, and traumatic injury (Medicinal Materials Company in Yunnan Province, 1993; Wu et al., 2015). Over the years, modern pharmacological research has shown that *A. zerumbet* has important physiological and pharmacological functions, including antioxidative, antimicrobial, antianxiety effects, and promotes osteoblastic cell differentiation activities (Elzaawely et al., 2007; Sousa et al., 2015; Kumagai et al., 2016; Xuan et al., 2016; Castro et al., 2018). Meanwhile, *A. galanga* has been used as an antifungal, antimicrobial, anti-inflammatory, antioxidant, and anti-osteoarthritic drug (Chouni and Paul, 2018). However, the medicinal market for *Alpinia* is chaotic, and source materials are often misidentified due to the similarities in morphological characters between *Alpinia* species and their medicinal organs; for example, the rhizomes of *A. galanga*, *A. calcarata*, and *A. officinarum* have been adulterated or substituted for one another during the sales process (Upadhye et al., 2018; Li J. Z. et al., 2020). These problems have severely hindered the clinical use of and scientific research related to medicinal *Alpinia* species.

*Alpinia* is the largest, most widespread genus in the Zingiberaceae, and it includes many cultivated varieties (Wu and Larsen, 2000; Zhao et al., 2001; Kress et al., 2005). This has led to substantial difficulties in the classification and identification of *Alpinia* species, and researchers have studied the molecular markers of species in this genus. Kress et al. (2002, 2005) conducted a thorough phylogenetic analysis of Zingiberaceae and *Alpinia* species using the DNA sequences of the internal transcribed spacer (ITS) and plastid *matK* regions, and the results indicate that *Alpinia* is a complex polyphyletic group. A phylogenetic analysis of Zingiberaceae plants, conducted based on the *ycf1* barcode, could not accurately classify taxa below the genus level (Zhong et al., 2018). These results indicate that commonly used DNA barcoding sequences are only useful for genus-level identification, and the relationships among species within the genus remain challenging to determine.

Chloroplasts (CPs) are important organelles in plant cells, as they are the location of photosynthesis. CPs convert solar energy into chemical energy and release oxygen while providing essential energy for the growth and reproduction of green plants (Wicke et al., 2011; Bock and Knoop, 2012). The CP genome consists of a closed circular DNA molecule that includes a large single-copy (LSC) region, a small single-copy (SSC) region, and two inverted repeats (IRa and IRb) (Sato et al., 1999; Ferrarini et al., 2013). CP genomes have been widely used for species evolution analysis, species identification, and the development of molecular markers because of their highly conserved gene sequences (Gao et al., 2019; Zhang et al., 2019; Li D. M. et al., 2020). With the rapid development of high-throughput sequencing technologies in recent years, the complete CP genome sequence has been obtained become easier. CP genomes have shown great potential for use in species identification, particularly closely related species (Park et al., 2018; Cui et al., 2019a). The complete CP genome, as a barcode has been widely used to evaluate plant phylogenetic relationships or distinguish species, and selected sequences from high-variation regions of the complete CP genome have been used for species identification (Chen et al., 2018; Cui et al., 2019a; Huo et al., 2019; Zhang et al., 2019). For example, Zhang et al. (2021) identified barcode markers to aid in the accurate identification of raw materials of Dragon’s blood (*Dracaena*) species in China by comparing the complete CP genome sequences of all species in the genus. In a recent study, a phylogenetic analysis of all genera in the Zingiberaceae was conducted using the complete CP genomes of three medicinal *Alpinia* species, and the phylogenetic relationship between *A. zerumbet* and *A. oxyphylla* was evaluated using the entire CP genome (Gao et al., 2019; Li D. M. et al., 2020). However, these publicly available CP genome data are insufficient to resolve the intraspecific and interspecific differences among *Alpinia* species, which exhibit only subtle morphological differences. Relative to the rest of the plant kingdom, a very limited amount of CP genome data is available for medicinal plants. It is therefore essential to obtain additional CP genome data to support the effective utilization of medicinal plant resources.

We sequenced the complete CP genomes of *A. galanga* and *A. kwangsiensis* sampled from Yunnan, using the Illumina HiSeq4000 sequencing platform. Next, we investigated their essential characteristics [including analyses of molecular structure, simple sequence repeats (SSRs), and long repeats]. Then, we compared the resulting CP genomes with the published CP genomes of *A. hainanensis* (MK262728), *A. oxyphylla* (MK262729), *A. pumila* (MK262731) (Gao et al., 2019; Li D. M. et al., 2020), and *A. zerumbet* (JX088668), and found potential high-variation region markers for *Alpinia*. Finally, we collected 28 whole CP genome sequences for Alpinieae species, which we used as a super-barcode to identify species in this group and analyze their phylogenetic positions. Our study provides significant genetic information for species identification and phylogenetic analyses of *Alpinia* plants. In addition, it can serve as a reference to help alleviate issues with the accurate identification of *Alpinia* plants in the medicinal market.

## Materials and Methods

### Plant Materials and DNA Extraction

Fresh *A. galanga* and *A. kwangsiensis* leaves were collected from Xishuangbanna City, Yunnan Province. Voucher specimens were deposited in the Yunnan branch of the Institute of Medicinal Plant Development (IMPLAD), Chinese Academy of Medical Sciences herbarium. The fresh leaves were cleaned with 75% ethanol and preserved at −80°C. Total genomic DNA was extracted from frozen clean leaves using the TaKaRa Mini BEST Universal Genomic DNA Extraction Kit with a standard protocol (TaKaRa, Shiga, Japan). The concentration and quality of DNA were checked using electrophoresis in a 1% (w/v) agarose gel and the Nanodrop 2000 instrument (Thermo Scientific, Waltham, MA, United States). The OD260/280 values ranged from 1.8 to 2.2, and ≥2 μg of DNA was equally pooled from individuals of the two species to construct a shotgun library.

### Chloroplast Genome Sequencing and Assembly

DNA samples were randomly sheared, incubated with fragmentation buffer, and broken into 300–500-bp fragments in a Covaris M220 focused ultrasonicator (Covaris, Woburn, MA, United States). The DNA library was prepared using the Illumina TruSeq™ Nano DNA Sample Prep Kit (Illumina, San Diego, CA, United States). To complement the fragmented DNA ends, A was added to the 3′ end of double-stranded DNA to form a sticky end, and a sequence adapter index was added. Polymerase chain reaction (PCR) amplification was performed for eight cycles for library enrichment, and the target band was recovered from a 2% agarose gel (Certified Low Range Ultra Agarose). Bridge PCR amplification was performed with the cBot solid phase (Truseq PE Cluster Kit v3-cBot-HS; Illumina) carrier to generate clusters. The library was sequenced at the Biozeron Company (Shanghai, China) using the Illumina HiSeq4000 sequencing platform to obtain 2 × 150 bp paired-end reads. Raw reads were checked, first using FastQC (Brown et al., 2017) and then using Trimmomatic version 0.39 (Bolger et al., 2014). Adapter sequences in the reads were removed, and low-quality reads were filtered from the raw data. Reads containing 10% N were removed, and small fragments of <75 bp were discarded after quality pruning. The CP genomes of *A. hainanensis* (MK262728), *A. oxyphylla* (MK262729), and *A. pumila* (MK262731), which are closely related species, were used as reference genomes. We used NOVOPlasty version 2.7.2^1^ (Dierckxsens et al., 2017) to assemble a contig containing the complete CP genome sequence. Then, using Celera Assembler version 8.0 (Denisov et al., 2008), we assembled and cleaned the data and constructed scaffolds of the CP genome using SSPACE (Boetzer et al., 2011). We optimized the assembly results using GapCloser version 1.12 (Luo et al., 2012), which repairs gaps. Finally, we used the reference genome to correct the starting position of the CP assembly sequence and determine the position and direction of the four CP regions (LSC, IRa, SSC, and IRb), to generate the assembled CP genomic sequence.

### Chloroplast Genome Annotation and Structure Analysis

We used the online tool Dual Organellar GenoMe Annotator (DOGMA; University of Texas at Austin, Austin, TX, United States) (Wyman et al., 2004) and the Chloroplast Genome Annotation Software with manual corrections to perform preliminary gene annotation of the CP genomes of both species of interest. We performed a BLASTN search on the National Center for Biotechnology Information (NCBI) website to identify and confirm boundary junctions, introns, exons, and coding regions. The tRNA genes were further verified using tRNAscanSE (Lowe and Chan, 2016) and DOGMA (Wyman et al., 2004) with the default settings. Additionally, the online OrganellarGenomeDRAW (OGDRAW) software version 1.2 (Max Planck Institute of Molecular Plant Physiology, Potsdam, Germany) (Greiner et al., 2019) was used to construct the gene map using the default settings and manual checking. Finally, we generated an.sqn file to submit our findings to NCBI. The high-quality reads were deposited into the NCBI BioProject database. The complete and correct CP genome sequences of *A. galanga* and *A. kwangsiensis* were deposited in GenBank under the accession numbers MZ066611 and MZ066612, respectively. CodonW software (University of Texas, Houston, TX, United States) with the relative synonymous codon usage (RSCU) ratio were used to investigate the codon distribution (Sharp and Li, 1987). The guanine–cytosine (GC) content was analyzed using Molecular Evolutionary Genetics Analysis Version X (Kumar et al., 2018).

### Repeat Sequence Analysis

Simple sequence repeats were detected using MISA software^2^, with the parameters set to encompass mononucleotide SSRs with ≥10 repeat units, di- and tri-nucleotide SSRs with ≥5 and 4 repeat units, respectively, and tetra-, penta-, and hexanucleotide SSRs with ≥3 repeat units. We identified the size and location of repeat sequences in the CP genomes of *A. galanga* and *A. kwangsiensis* using REPuter (University of Bielefeld, Bielefeld, Germany) (Kurtz et al., 2001) with the following parameters: 90% similarity percentage of scattered repeat copies and a minimum repeat size of 30 bp.

### Genome Comparison Analyses and Marker Discovery

The whole CP genomes were initially aligned using the online MAFFT software (Katoh et al., 2019). Conserved sequences between the CP genomes of *A. galanga* and *A. kwangsiensis* were identified using BLASTN with an E-value cutoff of 1e-10. The mVISTA (Frazer et al., 2004) program in Shuffle-LAGAN mode was used to compare the two *Alpinia* CP genomes using the *A. galanga* CP genome as a reference. Then, we used DnaSP (Rozas et al., 2017) software to determine the nucleotide variability (Pi) with a 200 bp step size and a 600 bp window length. Ten highly variable sites (*psbK-psbI*, *trnS-trnG*, *trnC-petN*, *rps4-trnT*, *rpl32-trnL*, *psaC-ndhE*, *ndhC-trnV*, *ndhF-rpl32*, *trnT-trnL*, and *psbE-petL*) were selected. We used the primer design tool Primer-BLAST to design labeled primers for the highly variable regions (Ye et al., 2012). Next, a total of seven *Alpinia* species (*A. zerumbet*, *A. galanga*, *A. blepharocalyx*, *A. kwangsiensis*, *A. hainanensis*, *A. conchigera*, and *A. oxyphylla*) (Supplementary Table 1) were used to assess the identification efficiency of the highly variable sites. Total genomic DNA was extracted using the TaKaRa MiniBEST Universal Genomic DNA Extraction Kit with a standard protocol (TaKaRa) and 1% agarose gel electrophoresis. We used an ultra-micro ultraviolet spectrophotometer to assess the purity and concentration of the extracted genomic DNA. The PCR reactions were conducted in a total reaction volume of 25 μL, which contained DNA (0.5 μL), 10 × PCR buffer (2.5 μL), dNTPs (2.5 mM, 2 μL), primers (0.5 μL each), Taq DNA polymerase (5 U/μL, 0.5 μL; TaKaRa), and double-distilled water (18.5 μL). For each reaction, we used the following program: an initial 5 min of denaturation at 94°C; 35 cycles of 30 s at 94°C, 30 s of annealing at Tm with different primers, and 15 s of extension at 72°C; and a final extension for 7 min at 72°C. The PCR products were visualized using 2% agarose gels, and the successfully amplified PCR products were sent to Sangon Biotech (Shanghai, China) for bidirectional sequencing. Finally, we used high-quality sequences to construct neighbor-joining (NJ) phylogenetic trees.

### Phylogenetic Analyses

To determine the phylogenetic positions of *A. galanga* and *A. kwangsiensis*, we downloaded 28 complete CP genomes of Alpinieae species from the NCBI database. The sequences were initially compared using MAFFT (Katoh et al., 2019). Next, we conducted multiple sequence visual analyses and manually delete useless gaps using BioEdit (Hall, 1999). We also used the CP genomes of *Curcuma longa* (MK262732) and *Zingiber officinale* (NC_044775) as outgroups. We constructed phylogenetic trees with 32 CP genomes sequences using the NJ, maximum parsimony (MP) and maximum likelihood (ML) methods with MEGA X software and 1000 bootstrap replicates (Kumar et al., 2018). The best-fit substitution models were selected by ModelTest-NG (Darriba et al., 2019) for ML trees.

## Results and Discussion

### Chloroplast Genome Features of *Alpinia* Species

**FIGURE 1:**
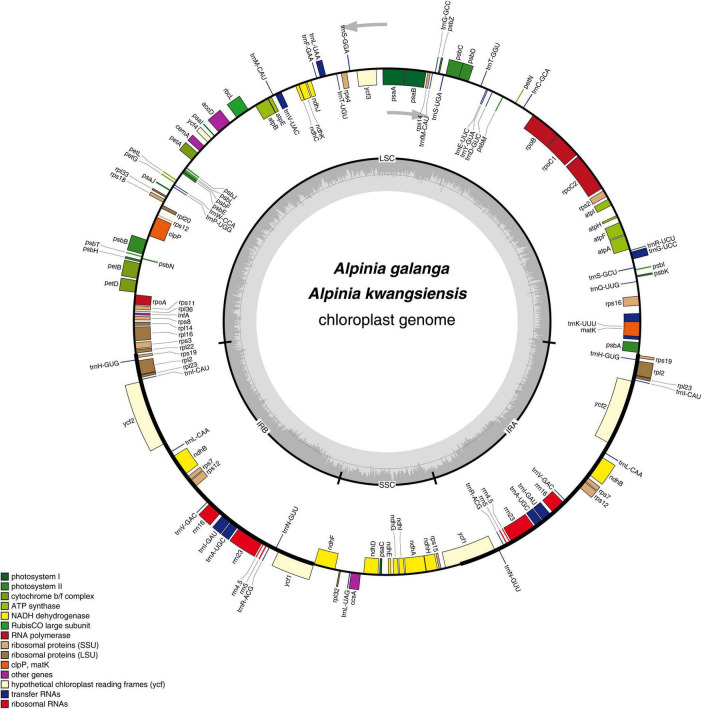
Gene map of the *Alpinia* complete chloroplast (CP) genome. Genes inside and outside the circle are transcribed clockwise and counterclockwise, respectively. Genes belonging to different functional groups are indicated by different colors. The darker gray area in the inner circle corresponds to the GC content, whereas the lighter gray area corresponds to the AT content.

The complete CP genome sequences of *A. galanga* and *A. kwangsiensis* are 160,100 and 162,323 bp in length, respectively, with both having an obvious quadripartite structure (Figure 1). The whole CP genomes contain a pair of IRs (IRa and IRb) at, respectively, 27,313 and 29,705 bp separated by an SSC region at 17,909 and 15,181 bp, and a LSC region at 87,565 and 87,732 bp. The *A. kwangsiensis* CP genome (162,323 bp) is 2223 bp longer than that of *A. galanga*. The GC contents of the CP genomes of *A. galanga* and *A. kwangsiensis* are 36.26 and 36.15%, respectively. And the GC content is unevenly distributed across different regions of each CP genome. The GC content in the IR regions is the highest (41.2–42.2%), followed by the LSC region at ∼33.8–33.9%, and the lowest content was found in the SSC region (29.8–30.0%). Moreover, the AT content at the third codon position (71.2–71.4%) is higher than that at the second (62.2–62.6%) and first (55.2–55.4%) positions in the protein-coding genes of the two *Alpinia* species (Table 1). In the complete CP genomes of both *Alpinia* species, 133 genes were detected, including 87 distinct protein-coding genes, eight distinct rRNA genes, and 38 distinct tRNA genes (Table 2). The distribution of genes in the two CP genomes is the same: 81 genes are distributed in the LSC region, including 60 protein-coding genes and 21 tRNA genes, whereas the SSC region contains 11 protein-coding genes and one tRNA gene; a total of 20 genes are duplicated in the IR regions, including eight protein-coding genes, eight tRNA genes, and four rRNAs (Figure 1 and Supplementary Table 2). These genomic structure features are similar to those of the other published CP genomes of the family Zingiberaceae (Cui et al., 2019a; Gao et al., 2019; Li D. M. et al., 2020).

**TABLE 1:** The base composition of the *A. galanga* and *A. kwangsiensis* CP genomes.

Region	*A. galanga*					*A. kwangsiensis*				
	T (U) (%)	C (%)	A (%)	G (%)	Length (bp)	T (U) (%)	C (%)	A (%)	G (%)	Length (bp)
Total	32.3	18.4	31.5	17.8	160100	32.2	18.4	31.6	17.8	162323
IRa	29.0	21.9	28.9	20.3	27313	30.0	21.3	28.8	19.9	29705
IRb	28.9	20.3	29.0	21.9	27313	28.8	19.9	30.0	21.3	29705
LSC	33.8	17.3	32.3	16.6	87565	33.8	17.3	32.4	16.5	87732
SSC	35.3	15.6	34.9	14.2	17909	34.0	15.9	36.0	14.1	15181
CDS	31.7	17.2	31.2	19.9	80775	31.6	17.2	31.5	19.7	83001
First position	24.0	18.3	31.2	26.6	26925	23.9	18.2	31.5	26.3	27667
Second position	32.5	20.1	29.7	17.6	26925	32.5	20.0	30.1	17.4	27667
Third position	38.5	13.3	32.7	15.5	26925	38.4	13.2	33.0	15.4	27667

**TABLE 2:** Genes present in the CP genomes of two *Alpinia* species.

Group of genes	Gene names	Amount
Photosystem I	*psaA*, *psaB*, *psaC*, *psaI*, and *psaJ*	5
Photosystem II	*psbA*, *psbB*, *psbC*, *psbD*, *psbE*, *psbF*, *psbH*, *psbI*, *psbJ*, *psbK*, *psbL*, *psbM*, *psbN*, *psbT*, and *psbZ*	15
Cytochrome b/f complex	*petA*, petB*, petD*, *petG*, *petL*, and *petN*	6
ATP synthase	*atpA*, *atpB*, *atpE*, atpF*, *atpH*, and *atpI*	6
NADH dehydrogenase	ndhA*, ndhB*(× 2), *ndhC*, *ndhD*, *ndhE*, *ndhF*, *ndhG*, *ndhH*, *ndhI*, *ndhJ*, and *ndhK*	12
RubisCO large subunit	*rbcL*	1
RNA polymerase	*rpoA*, *rpoB*, rpoC1*, and *rpoC2*	4
Ribosomal proteins (SSU)	*rps2*, *rps3*, *rps4*, *rps7*(× 2), *rps8*, *rps11*, rps12*(× 2), *rps14*, *rps15*, rps16*, *rps18*, and *rps19*(× 2)	15
Ribosomal proteins (LSU)	rpl2*(× 2), *rpl14*, rpl16*, *rpl20*, *rpl22*, *rpl23*(× 2), *rpl32*, *rpl33*, and *rpl36*	11
Other genes	*accD*, clpP**, *matK*, *ccsA*, *cemA*, and *infA*	6
Proteins of unknown function	*ycf1(*× *2)*, *ycf2*(× 2), ycf3**, and *ycf4*	6
Transfer RNAs	38 *tRNA*s (6 contain an intron, 8 in the IRs)	38
Ribosomal RNAs	*rrn4.5*(× 2), *rrn5*(× 2), *rrn16*(× 2), and *rrn23*(× 2)	8

*Gene containing one intron. **Gene containing two introns; (× 2) gene with two copies.

We combined the CP genome information of *A. galanga* and *A. kwangsiensis* with four CP genomes for *Alpinia* species published in the NCBI database and performed comparisons and analyses (Table 3). The results showed that the CP genome size in the six species varied from 159,773 bp (*A. zerumbet*) to 162,387 bp (*A. hainanensis*). Moreover, the LSC, SSC, and IR regions of the different species exhibited different characteristics. There were some interesting features; for example, *A. kwangsiensis* contained the longest LSC region (87,732 bp) but the shortest SSC region (15,181 bp), whereas *A. zerumbet* had the longest SSC region (18,295 bp) but the shortest IR (26,917 bp). In terms of the number of genes, except for *A. zerumbet* (which contained 132 genes), the remaining five species contained 133 genes; *A. zerumbet* lacked one protein-coding gene. Among the six species analyzed, the *A. zerumbet* CP genome had the highest GC content (36.27%), whereas the *A. hainanensis* and *A. kwangsiensis* CP genomes had the lowest GC content (36.15%).

**TABLE 3:** Comparison of the general features of the six *Alpinia* CP genomes.

Genome characteristics	*A. galanga*	*A. kwangsiensis*	*A. hainanensis*	*A. oxyphylla*	*A. pumila*	*A. zerumbet*
GenBank number	MZ066611	MZ066612	MK262728	MK262729	MK262731	JX088668
Genome size (bp)	160100	162323	162,387	161,410	161,920	159,773
LSC length (bp)	87565	87732	87,667	87,279	87,261	87,644
SSC length (bp)	17909	15181	15,306	16,180	15,317	18,295
IR length (bp)	27313	29705	29,707	28,964/28,987	29,671	26,917
Total genes	133	133	133	133	133	132
Protein-coding genes	87	87	87	87	87	86
tRNA genes	38	38	38	38	38	38
rRNA genes	8	8	8	8	8	8
GC content (%)	36.26	36.15	36.15	36.16	36.17	36.27

### Codon Usage

**FIGURE 2:**
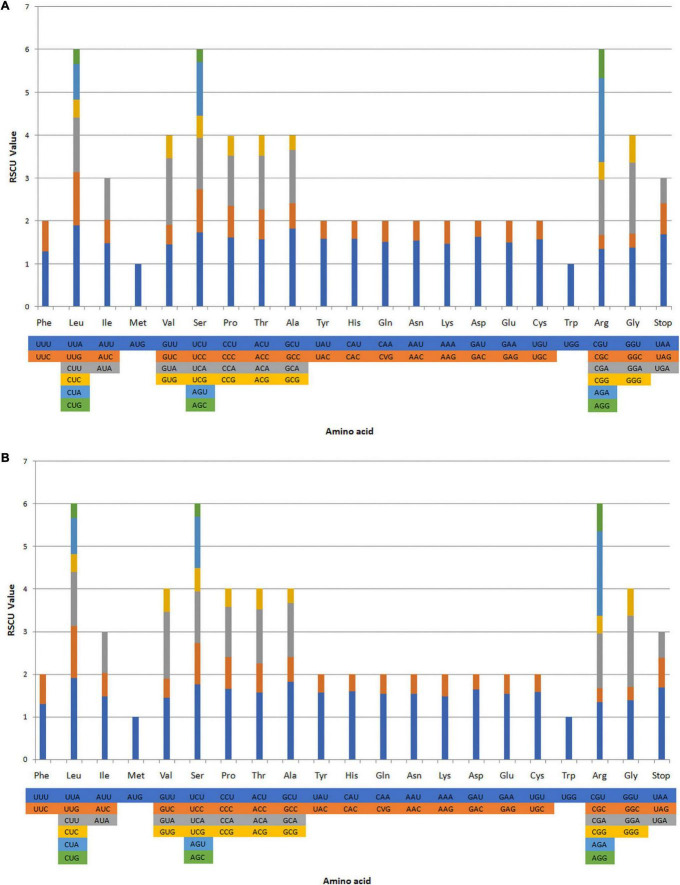
Codon contents in all protein-coding genes in the *A. galangal* **(A)** and *A. kwangsiensis* **(B)** complete chloroplast (CP) genome. RSCU, relative synonymous codon usage.

The RSCU ratio is a measure of uneven usage of synonymous and non-synonymous codons in a coding sequence. An RSCU ratio <1.00 indicates that the frequency of codon usage is lower than expected, whereas codons used more frequently than expected with a ratio >1.00 (Sharp and Li, 1987; Liu et al., 2018). The codon usage levels of the *A. galanga* and *A. kwangsiensis* CP genomes are shown in Figure 2 and Supplementary Table 3. We analyzed the codon usage frequency and RSCU ratios of 87 protein-coding genes in the *A. galanga* and *A. kwangsiensis* CP genomes. In total, the genes in the *A. galanga* and *A. kwangsiensis* CP genomes contain 26,925 and 27,667 codons, respectively. The codons for leucine, serine, and arginine are the most common in both the *A. galanga* and *A. kwangsiensis* CP genomes. In the CP genomes of these *Alpinia* species, usage of the codons AUG and UGG-encoding methionine and tryptophan, respectively-is not biased (RSCU ratio = 1.00). Usage of the UCC codon encoding serine is also not biased (RSCU ratio = 1.00) in the *A. galanga* CP genome. Most protein-coding genes in the CP genomes of terrestrial plants use standard AUG initiator codons, and the use of AUG to encode methionine is not biased (RSCU ratio = 1) in the *A. galanga* and *A. kwangsiensis* CP genomes. Codons ending in A and/or U account for 71.1 and 71.4% of all the protein-coding genes in the CP genomes of *A. galanga* and *A. kwangsiensis*, respectively. Codons ending in A and/or T (U) usually have high RSCU ratios in the two CP genomes, e.g., AGA (1.96) encoding arginine, GCU (1.82) encoding alanine, and UCU (1.74) encoding serine. These results are similar to those observed for *Zingiber officinale* and *Wurfbainia vera* (Wu et al., 2017; Cui et al., 2019b). The codon usage pattern can be determined by whether there is a high proportion of A/T component bias. In the CP genomes of other higher terrestrial plants, preferred codon usage tends to be very high, and the preference for A/T is widespread (Kim and Lee, 2004; Qian et al., 2013). Our results also showed that except for Leu-UUG, all types of preferred synonymous codons (RSCU ratio > 1.00) in the two *Alpinia* species end with A or U. The high RSCU ratio may be attributed to the functions of the amino acids or the structures of peptides needed to avoid transcription errors (Li et al., 2016; Gao et al., 2019). This phenomenon indicates that stable CP genome evolution helps to protect important CP genes from harmful mutations while improving adaptation to selection pressure (Wang et al., 2016; Ivanova et al., 2017; Zuo et al., 2017).

### Analyses of Simple Sequence Repeats and Long Repeats

**FIGURE 3:**
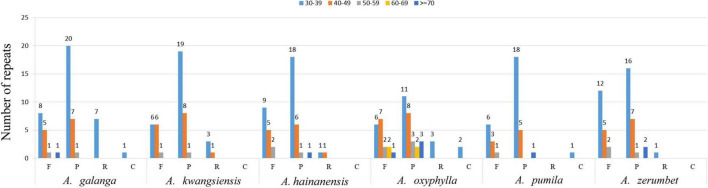
Long repeat sequence analysis of six *Alpinia* complete chloroplast (CP) genomes. REPuter was used to identify repeat sequences ≥30 bp in length and sequences with ≥90% similarity in the CP genomes. F, P, R, and C indicate the forward, palindromic, reverse, and complementary repeat types, respectively. Repeats with different lengths are indicated by different colors.

Simple sequence repeats, or microsatellites, are tandem repeat sequences consisting of 1–6 nucleotide repeat units and are widely distributed in CP genomes (Wu et al., 2017; Zhou J. et al., 2018). Repeated sequences were divided into tandem repeats and scattered repeats. Scattered repeats can be further divided into four types of repeats: complementary, forward, reverse, and palindromic (Kurtz et al., 2001; Zhang et al., 2019). These repeat structures promote intermolecular recombination and create diversity among CP genomes in the population (Guo et al., 2017; Zhou J. et al., 2018). In this manuscript, we used the Tandem Repeats Finder and REPuter software tools to analyze the repeat sequences and distribution of repeat sequences and SSRs in the CP genomes of six species (*A. galanga*, *A. kwangsiensis*, *A. hainanensis*, *A. oxyphylla*, *A. pumila*, and *A. zerumbet*). Results of the repeat-sequence structural analysis are shown in Figure 3. The results showed that the number of repeat types was very similar among the six *Alpinia* species; palindromes (24–28) were the most abundant, followed by forward (10–19) and reverse repeats (0–7), and complementary (0–2) repeats were the least abundant. In all six species, most of these repeats are between 30–39 and 40–49 bp in length, with only a few repeats >70 bp in length.

Furthermore, we analyzed the distribution and types of SSRs contained in the CP genomes of the six *Alpinia* species. We identified 110, 125, 113, 122, 121, and 118 SSRs in the CP genomes of *A. galanga*, *A. kwangsiensis*, *A. hainanensis*, *A. oxyphylla*, *A. pumila*, and *A. zerumbet* using MISA software, respectively (Table 4). The SSRs are mainly distributed in the LSC region of the CP genome, followed by the SSC region and the IR region (Supplementary Table 4). The most abundant type is repeated mononucleotides (50–60.17%), which were found 15–16 times in the six *Alpinia* species. These are followed by dinucleotide (20.03–30.33%), trinucleotide (3.28–5.31%), tetranucleotide (13.22–16.36%), and pentanucleotide repeats (0–3.64%). The A/T (46.36–57.63%) repeat is the most abundant motif in all repeats, followed by AT/TA (20.34–28.68%) dinucleotide repeats and AAAT/ATTT (5.93–8.18%) tetranucleotide repeats. Our results are consistent with previous studies reporting that CP SSRs usually consist of short poly-A or poly-T repeats. A and T are always the most frequently used bases, and tandem G or C repeats are rare in many plants (Kuang et al., 2011; Zhang et al., 2019). Interestingly, except for *A. galanga*, *A. kwangsiensis*, *A. oxyphylla*, and *A. pumila*, the other two species have no pentanucleotide SSRs, and none of the *Alpinia* species have any hexanucleotide SSRs. Because CP SSRs have high substitution rates, SSR markers are widely used in genetic diversity and population structure assessments, comparative genomics, the development of genetic maps, and marker-assisted selective breeding (Flannery et al., 2006; Chen et al., 2015; Cui et al., 2019b; Zhang et al., 2019). The repeat sequences identified in this study are a valuable resource for species identification as well as research on the genetic diversity and population structure of Zingiberaceae plants.

**TABLE 4:** The simple sequence repeat (SSR) types of the six CP genomes of *Alpinia* species.

SSR type	Repeat unit	Amount					
		*A. galanga*	*A. kwangsiensis*	*A. hainanensis*	*A. oxyphylla*	*A. pumila*	*A. zerumbet*
Mono	A/T	51	67	60	62	66	68
	C/G	4	2	0	1	2	3
Di	AG/CT	2	2	2	2	2	2
	AT/AT	26	31	27	35	30	24
Tri	AAG/CTT	3	3	3	3	2	3
	ACT/AGT	1	0	0	0	1	0
	AGG/CCT	1	1	1	1	1	1
	AAT/ATT	0	1	2	0	0	1
Tetra	AAAC/GTTT	1	1	1	1	1	1
	AAAG/CTTT	3	3	3	3	2	3
	AAAT/ATTT	9	8	9	9	8	7
	AACT/AGTT	1	1	1	1	1	1
	AATG/ATTC	1	2	2	1	2	2
	AATT/AATT	2	1	1	1	1	1
	ACAT/ATGT	1	1	1	1	1	1
Penta	AAAAT/ATTTT	1	0	0	0	0	0
	AATAT/ATATT	3	0	0	0	0	0
	AAGGT/ACCTT	0	1	0	0	0	0
	AAATT/AATTT	0	0	0	1	0	0
	AACAT/ATGTT	0	0	0	0	1	0

### Genome Comparison and Nucleotide Diversity

**FIGURE 4:**
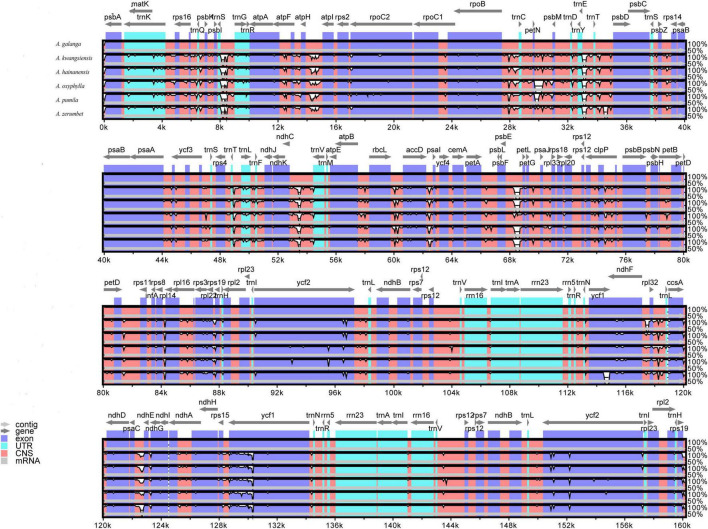
Structural comparison of the complete CP genomes of six *Alpinia* species using mVISTA. The CP genome of *A. galanga* was used as a reference. Gray arrows and thick black lines above the alignments indicate gene orientations and the positions of IRs in the genes, respectively. White peaks represent differences among CP genomes. A similarity cut-off value of 70% was used for the plots, and the *Y*-axis represents the percentage similarity (50–100%).

A comparison of CP genomes helps with elucidating the genetic structure and evolutionary relationships of plants in different environments (Daniell et al., 2016; Zhang et al., 2019). To determine the level of sequence similarity and genome rearrangements, we used mVISTA software to compare and analyze the sequence homologies of the CP genomes of six *Alpinia* species with *A. galanga* as the reference sequence (Figure 4). These analyses revealed few differences among the CP genomes of the six *Alpinia* species. These differences were mostly found in non-coding regions, such as *trnG-UCC*, *rps4-trnT*, *psaC-ndhE*, *rpl32-trnL-UAG*, *trnC-GCA-petN*, *trnS-GCU-trnG-UCC*, *ndhC-trnV*, and *psbK-psbI*. The most divergent coding regions include *rpoC2*, *ycf1*, *ycf2*, *atpE*, and *rpl22*. Furthermore, a comparison of the CP genomes of the six *Alpinia* species showed that most of the sequence variation is in the LSC and SSC regions, and the IR region exhibits the least sequence variation. This result further supports the view that the IR regions are more conserved than the LSC and SSC regions in higher plants (Nazareno et al., 2015; Cui et al., 2019a). Some scholars believe that this may be because gene conversion has corrected the mutations in the IR sequences (Khakhlova and Bock, 2006).

**FIGURE 5:**
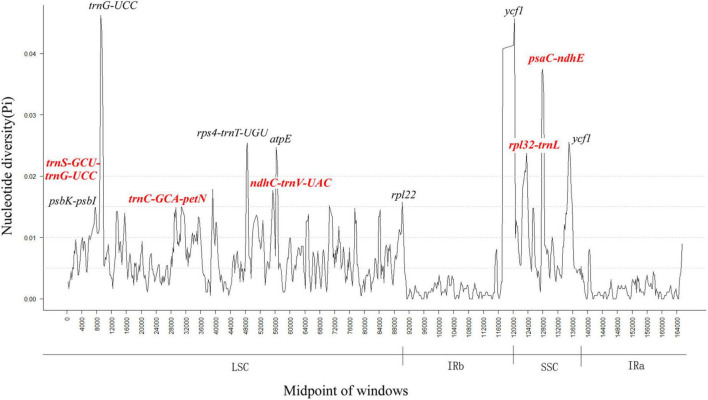
Sliding window analysis based on the complete chloroplast (CP) genomes of six *Alpinia* species. Window length: 600 bp; step size: 200 bp. *X*-axis: position of the midpoint of a window. *Y*-axis: nucleotide diversity of each window.

In addition, we also used DnaSP (Rozas et al., 2017) software to determine nucleotide diversity (PI) to detect sequence level differences in CP genomes of six *Alpinia* species and detecting highly variable regions (Figure 5). The IR region exhibits lower variability than the LSC and SSC regions, similar to previous studies (Zhou J. et al., 2018; Cui et al., 2019a; Li D. M. et al., 2020). The results showed an average value of Pi in all six *Alpinia* species of 0.0056 (Supplementary Table 5). In addition, some regions with high Pi values (>0.04) were observed in the LSC and SSC regions; for example, the Pi values of *trnG-UCC* and *ycf1* were 0.0462 and 0.0457, respectively. The results indicate that the LSC and SSC regions may be undergoing rapid nucleotide substitutions in Zingiberaceae species, and this variation plays an important role in species identification and phylogenetic analysis.

### Discovery of Candidate Markers Based on Highly Variable Regions

Previous molecular identification studies of *Panax*, *Zanthoxylum*, and *Gentiana* species showed that CP genetic markers had high identification capabilities (Lee et al., 2017; Nguyen et al., 2017; Zhou T. et al., 2018). Based on the alignment of complete CP genome sequences, 10 highly variable sites were selected to perform PCR amplification in seven species of *Alpinia*. Ultimately, five markers (*trnS-trnG*, *trnC-petN*, *rpl32-trnL*, *psaC-ndhE*, and *ndhC-trnV*) successfully amplified fragments of the expected sizes, and their PCR products were sent to the Sangon Laboratory for sequencing (Supplementary Table 6). An NJ phylogenetic tree was constructed using high-quality sequences to show the ability of the five highly variable regions in species-level identification (Supplementary Figure 1), using 50% as a cut-off value for the condensed tree. Yang et al. (2021) recently published an article on the development of molecular markers for five medicinal *Alpinia* species based on complete plastome sequences and developed molecular markers based on two highly variable regions (*petN-psbM* and *psaJ-rpl33*). We identified different highly variable sites from those of Yang et al. (2021) possibly because we used different *Alpinia* species in our analyses. And adding more species might be more accurate for finding and developing highly variable markers. In recent years, numerous studies have used plastid genomes to detect regions with high variation that may be used as molecular markers for species authentication (Jiang et al., 2017; Manzanilla et al., 2018; Zhou T. et al., 2018; Zhou Y. et al., 2018); however, this method is still limited to a few taxa. For example, Cui et al. (2019a) conducted a comparison and phylogenetic analysis of the CP genomes of *Wurfbainia* (= *Amomum*) species and identified four potential highly divergent region markers, but none provided sufficient discriminatory power to distinguish the eight study species. We infer that the reason for this result may be due to the slow evolution and monophyletic inheritance of cpDNA, so there are certain limitations in using cpDNA among species with frequent gene exchanges. *Alpinia* is an extremely complex group, and the method of using only a few species to find the identification markers for the genus remains to be verified. In summary, we used CP genome screening to detect highly variable regions that may be used as molecular markers for the authentication of *Alpinia* species, but their effectiveness of accurate identification remains to be further determined for the complex relationships within *Alpinia*.

### Phylogenetic Relationships of *Alpinia* Species

**FIGURE 6:**
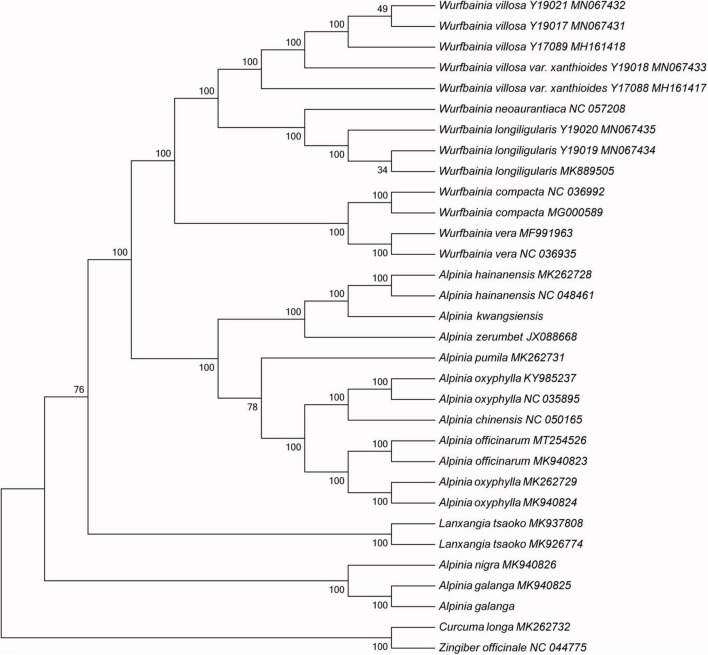
Phylogenetic tree constructed using the neighbor-joining method based on the 32 complete chloroplast (CP) genomes. Numbers at branch nodes are the bootstrap support values.

The CP sequence is essential for studying phylogenetic relationships and species identification and for determining taxonomic status in angiosperms (Li et al., 2015, 2019; Zhang et al., 2019). Alpinieae is the largest tribes of Zingiberaceae, including 25 genera. We obtained 28 complete CP genome sequences from NCBI for Alpinieae species (*Alpinia*, *Wurfbainia*, *and Lanxangia*). *C. longa* and *Z. officinale* (Zingibereae) was used as an outgroup for the construction of the NJ (Figure 6), MP (Supplementary Figure 2), and ML (Supplementary Figure 3) phylogenetic trees. The results of the NJ, MP, and ML phylogenetic trees further validate the phylogenetic relationships in the Zingiberaceae reported in previous studies (Kress et al., 2002, 2005; Meng et al., 2019). The structure of these phylogenetic trees shows the close relationships among species, with support values of >70%. *A. galanga* and *A. nigra* are the first to split off into a separate branch, whereas the remaining species are included in another large branch. Then, the two sequences of *Lanxangia tsaoko* split off into a branch, and the remaining *Alpinia* and *Wurfbainia* species split off into a large branch. Next, *Alpinia* and *Wurfbainia* species clustered into two branches, respectively. This result further proves the research conclusion of Kress et al. (2002) and de Boer et al. (2018) that *Alpinia* is a non-monophyletic genus: the genus *Alpinia* forms six polyphyletic clades (including clades II and IV in our study). *A. oxyphylla* (NC-035895 and KY985237) from Hainan is closer to *A. chinensis* and *A. oxyphylla* (MK262729)/*A. oxyphylla* (MK940824) from Guangdong/Guangxi is closer to *A. officinarum* in *Alpinia*. This result may be because the CP genome sequences of *A. oxyphylla* individuals representing different regions and varieties exhibit relatively obvious variations, resulting in greater intraspecific than interspecific variation in the CP genome of *A. oxyphylla*. Furthermore, we believe that the changes in the relationships between some species are due to addition of more samples therefore species relationships in this genus will be clearer when more species are included in the analyses. Due to the wide variety of *Alpinia* plants, the taxonomic status and phylogenetic relationships of many species have been difficult to determine, and future phylogenetic analyses should include more CP genome samples. Our results provide a valuable reference and a foundation for using CP genomes in species identification, and aid in improving the understanding of the phylogeny of *Alpinia* plants.

## Conclusion

We used high-throughput sequencing to sequence the complete CP genomes of *A. galanga* (160,100 bp) and *A. kwangsiensis* (162,323 bp), both of which exhibited an obvious quadripartite structure. We then combined these data with publicly available CP genome data for four species (*A. hainanensis*, *A. oxyphylla*, *A. pumila*, and *A. zerumbet*) and found five candidate highly variable marker sites (*trnS-trnG*, *trnC-petN*, *rpl32-trnL*, *psaC-ndhE*, and *ndhC-trnV*) based on the alignment of complete CP genome sequences. Next, we obtained existing CP genome sequences for Alpinieae from NCBI to construct phylogenetic trees and revealed limited phylogenetic relationships within the groups. The complete CP genomes of the two medicinal *Alpinia* species provides lays the foundation for the use of CP genomes in species identification and phylogenetic analyses of *Alpinia* species.

## Data Availability Statement

The original contributions presented in the study are publicly available. This data can be found here: The complete and correct CP genome sequences of *A*. *galanga* and *A*. *kwangsiensis* were deposited in GenBank under the accession numbers MZ066611 and MZ066612, respectively.

## Author Contributions

Z-LZ and YZ conceived and designed the manuscript. YZ, M-FS, C-YY, and H-FS analyzed the experiments data. YZ executed the manuscript. Z-LZ revised the manuscript. Z-LZ, YZ, D-YT, and A-SX collected the samples. L-XZ and YL provided technical support. All authors approved the final manuscript.

## Conflict of Interest

The authors declare that the research was conducted in the absence of any commercial or financial relationships that could be construed as a potential conflict of interest.

## Publisher’s Note

All claims expressed in this article are solely those of the authors and do not necessarily represent those of their affiliated organizations, or those of the publisher, the editors and the reviewers. Any product that may be evaluated in this article, or claim that may be made by its manufacturer, is not guaranteed or endorsed by the publisher.

## Footnotes

1
https://github.com/ndierckx/NOVOPlasty

2
http://pgrc.ipk-gatersleben.de/misa/
