# Supplementary material for: Complete Chloroplast Genome Analysis of Two Important Medicinal Alpinia Species: Alpinia galanga and Alpinia kwangsiensis
Source: Front Plant Sci. 2021 Dec 15;12:705892. doi: 10.3389/fpls.2021.705892 (PMC8714959; doi:10.3389/fpls.2021.705892)
Supplement: Supplementary file 1 [file Data_Sheet_1.pdf]

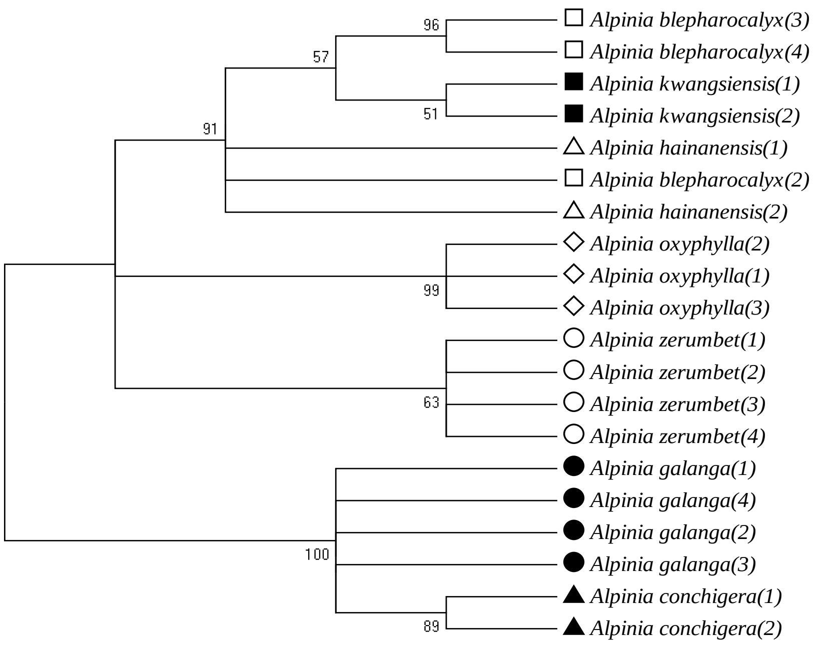

NJ phylogenetic tree of seven *Alpinia* species based on the concatenated sequences of *ndhC-trnV*.

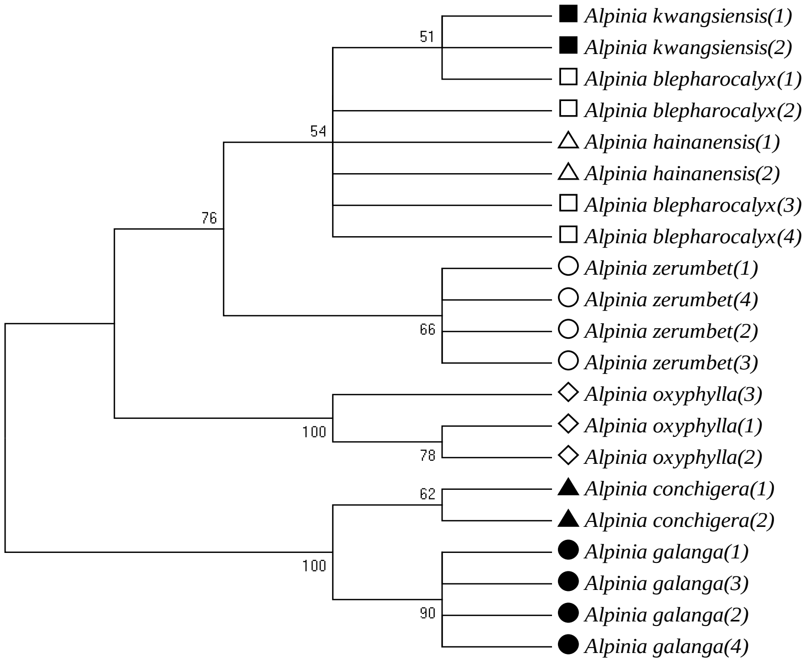

NJ phylogenetic tree of seven *Alpinia* species based on the concatenated sequences of *psaC-ndhE*.

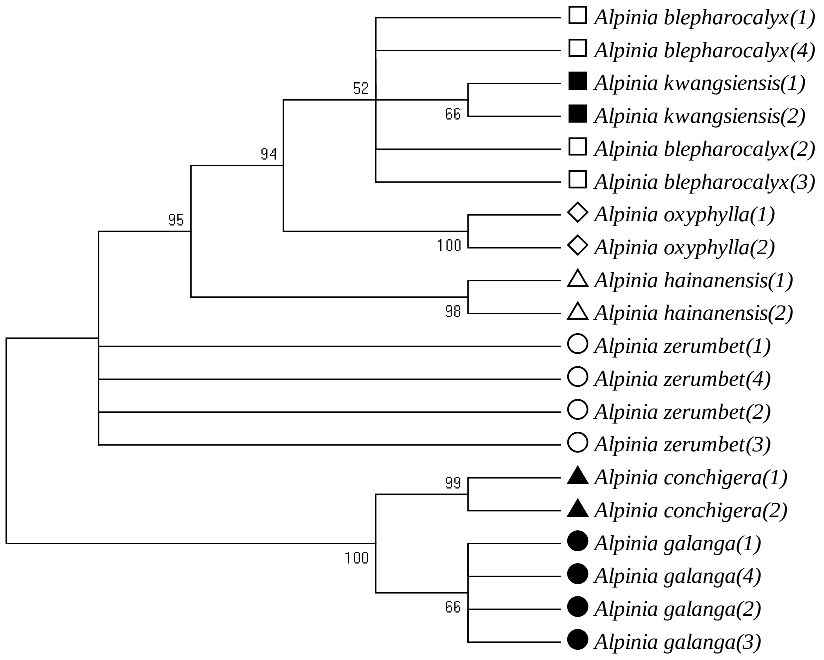

NJ phylogenetic tree of seven *Alpinia* species based on the concatenated sequences of *rpl32-trnL*.

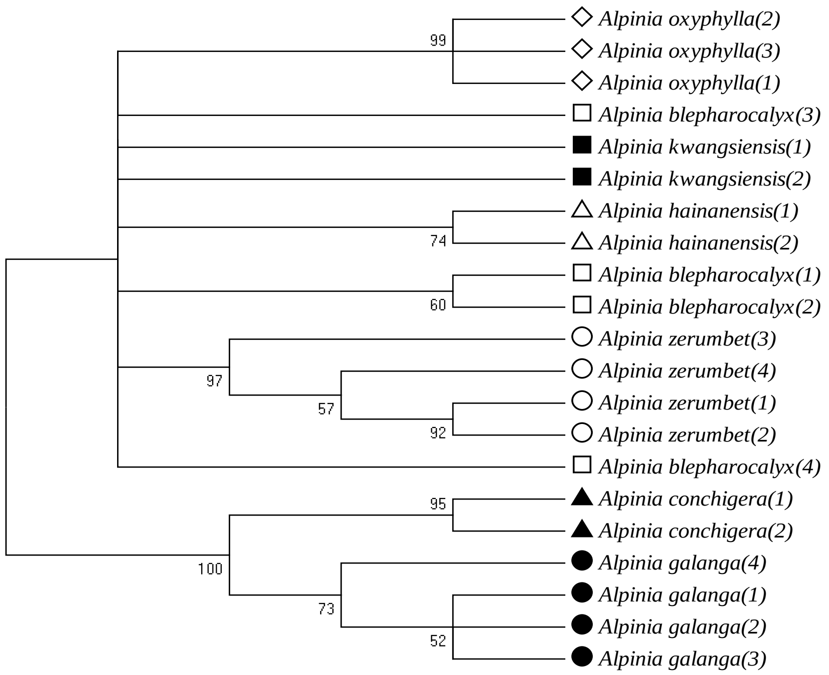

NJ phylogenetic tree of seven *Alpinia* species based on the concatenated sequences of *trnC-petN*.

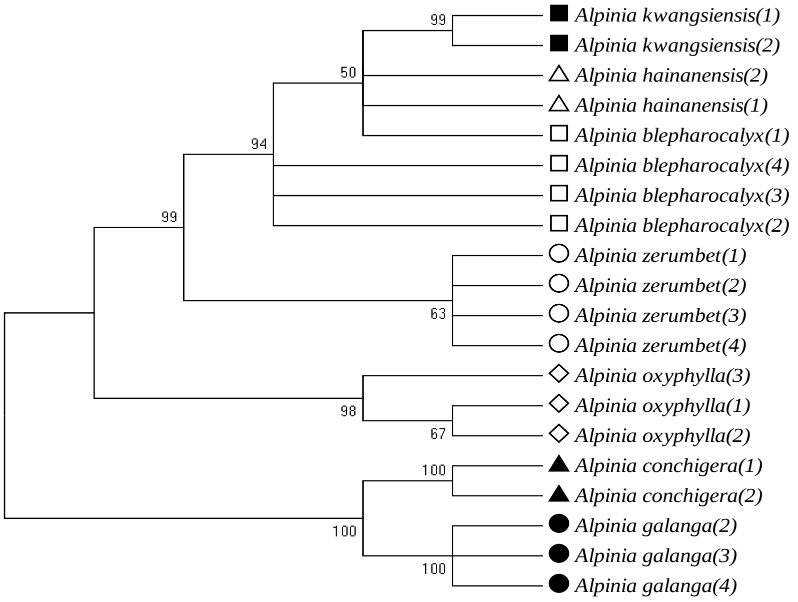

NJ phylogenetic tree of seven *Alpinia* species based on the concatenated sequences of *trnS-trnG*.

Supplementary Figure 1 Phylogenetic tree constructed using neighbor-joining based on the 5 Barcode Markers of seven *Alpinia* species. Numbers above the branches are the bootstrap support values.
