# Supplementary material for: Complete Chloroplast Genome Analysis of Two Important Medicinal Alpinia Species: Alpinia galanga and Alpinia kwangsiensis
Source: Front Plant Sci. 2021 Dec 15;12:705892. doi: 10.3389/fpls.2021.705892 (PMC8714959; doi:10.3389/fpls.2021.705892)
Supplement: Supplementary file 2 [file Data_Sheet_2.pdf]

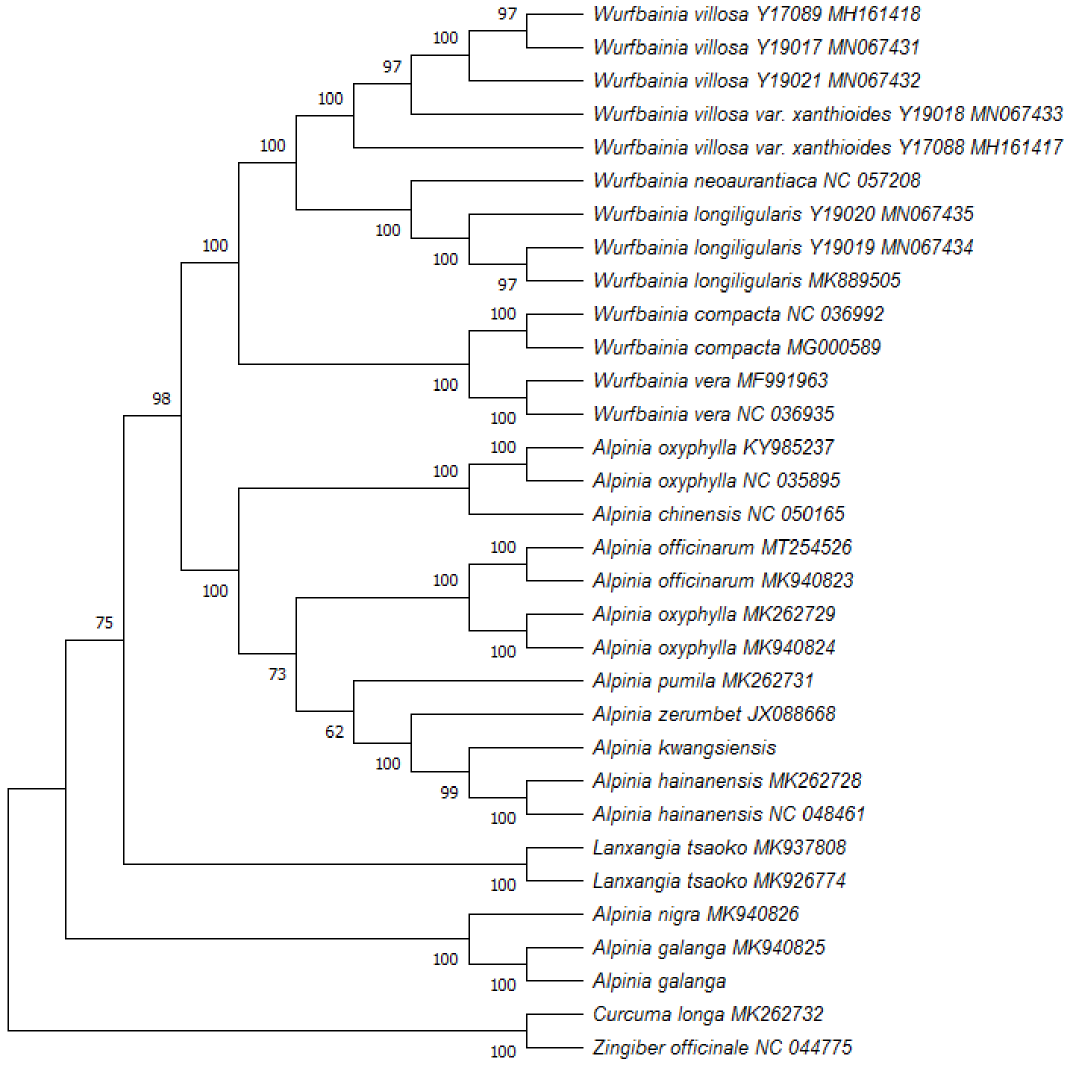

Supplementary Figure 2 Phylogenetic tree constructed using maximum parsimony (MP) based on the 32 complete CP genomes. Numbers above the branches are the bootstrap support values.
