# Supplementary material for: Complete Chloroplast Genome Analysis of Two Important Medicinal Alpinia Species: Alpinia galanga and Alpinia kwangsiensis
Source: Front Plant Sci. 2021 Dec 15;12:705892. doi: 10.3389/fpls.2021.705892 (PMC8714959; doi:10.3389/fpls.2021.705892)
Supplement: Supplementary file 4 [file Table_1.DOCX]

**Supplementary Table1** *Alpinia* species sample collection information

| **Species-No.** | **Location** | **acquisition time** |
| --- | --- | --- |
| *Alpinia zerumbet*(1) | Cangyuan,Yunnan Province,China | August, 2020 |
| *Alpinia zerumbet*(2) | Cangyuan,Yunnan Province,China | August, 2020 |
| *Alpinia zerumbet*(3) | Luchun,Yunnan Province,China | August, 2020 |
| *Alpinia zerumbet*(4) | Menglian,Yunnan Province,China | July,2020 |
| *Alpinia galanga*(1) | Luchun,Yunnan Province,China | August, 2020 |
| *Alpinia galanga*(2) | Jinghong,Yunnan Province,China | October, 2020 |
| *Alpinia galanga*(3) | Jinghong,Yunnan Province,China | October, 2020 |
| *Alpinia galanga*(4) | Yingjiang,Yunnan Province,China | August, 2020 |
| *Alpinia blepharocalyx*(1) | Luchun,Yunnan Province,China | August, 2020 |
| *Alpinia blepharocalyx*(2) | Luchun,Yunnan Province,China | August, 2020 |
| *Alpinia blepharocalyx*(3) | Menglian,Yunnan Province,China | July,2020 |
| *Alpinia blepharocalyx*(4) | Menglian,Yunnan Province,China | July,2020 |
| *Alpinia kwangsiensis*(1) | Jinghong,Yunnan Province,China | October, 2020 |
| *Alpinia kwangsiensis*(2) | Jinghong,Yunnan Province,China | October, 2020 |
| *Alpinia hainanensis* (1) | Yingjiang,Yunnan Province,China | August, 2020 |
| *Alpinia hainanensis* (2) | Yingjiang,Yunnan Province,China | August, 2020 |
| *Alpinia conchigera*(1) | Jinghong,Yunnan Province,China | October, 2020 |
| *Alpinia conchigera*(2) | Jinghong,Yunnan Province,China | October, 2020 |
| *Alpinia oxyphylla*(1) | Jinghong,Yunnan Province,China | October, 2020 |
| *Alpinia oxyphylla*(2) | Jinghong,Yunnan Province,China | October, 2020 |
| *Alpinia oxyphylla*(3) | Jinghong,Yunnan Province,China | October, 2020 |
