# Supplementary material for: Complete Chloroplast Genome Analysis of Two Important Medicinal Alpinia Species: Alpinia galanga and Alpinia kwangsiensis
Source: Front Plant Sci. 2021 Dec 15;12:705892. doi: 10.3389/fpls.2021.705892 (PMC8714959; doi:10.3389/fpls.2021.705892)
Supplement: Supplementary file 9 [file Table_6.docx]

**Supplementary Table6** The PCR amplification primer sequence and amplification program of five successfully amplified high-variability sites barcode

| **Regions** | **Primer pairs** | **Sequence 5´-3´** | **Thermocycling conditions** |
| --- | --- | --- | --- |
| *trnC-petN* | F | CGG AGA AGT ATA AGC CTA TCG | 94 ℃ 3 min; [35 cycles: 94 ℃ 60 s, 52 ℃/52 ℃/54 ℃/54 ℃/55 ℃ 30 s, 72 ℃ 80 s]; 72 ℃ 10 min |
|  | R | ACC ATT AAA GCA GCC CAA G |  |
| *psaC-ndhE* | F | AGA CAC CAG ACG AAG CA |  |
|  | R | CCG AAG CAT CTA TTG GAC T |  |
| *Rpl32-trnL* | F | AGG GTG TGT ATA TTG GGT TT |  |
|  | R | TAA GAG CAG CGT GTC TAC |  |
| *ndhC-trnV* | F | GGG TGC TCC TTC TTA CAA A |  |
|  | R | GAT TCC GTC GAT TAG ATT ACT C |  |
| *trnS-trnG* | F | TTA GTC CAC TCA GCC ATC T |  |
|  | R | GAG ACG CAA TCA AGA TAG GA |  |
